# 

**Published:** 1911-02-11

**Authors:** 


### BOOKS RECEIVED.

T. B. Browne, Ltd.
"A.B.C. of Advertisers, 1911."

Sanitary Publishing Co.
"*Sanitary Record* Year-book and Diary, 1911."

The Workmen's Committee of the Durham County Hospital, under the leadership of Mr. J. Beamson, has decided to raise the £400 necessary for the additional accommodation which the hospital requires.

### Gifts and Bequests.

The King has sent presents of game to the East London Hospital for Children, Shadwell, E., the Victoria Hospital for Children, Chelsea, the Royal Hospital for Incurables, Putney Heath, the Chelsea Hospital for Women, the Samaritan Hospital for Women, the Royal National Orthopædic Hospital, the Royal Ear Hospital, Soho, and to the Royal London Ophthalmic Hospital (Moorfields Eye Hospital).

Mrs. Elizabeth Doleman, of South Shore, Blackpool, has left £5 5s. per annum, payable for 15 years after her death, to the Bolton Infirmary, the Devonshire Hospital, Buxton, the Blackburn Infirmary, and to the Victoria Hospital, Blackpool.

### Jottings.

The new rule, that patients shall not leave the hospital for any purpose without the permission of the doctor in attendance, was passed at the annual meeting of the Tenby Cottage Hospital. Mr. G. E. Maitland, the Hon. Secretary, received a special vote of thanks for his work.

The Duke of Portland, presiding last Saturday at the annual meeting of the Queen Victoria Jubilee Institute for Nurses, referred to the annual debt of £2,500. He was glad they had been able to meet it this year, but the future had to be considered, and the council had appointed a committee.

### BUILDINGS CONTEMPLATED AND IN PROGRESS.

Buckhaven.—Plans by W. D. Telfer, Esq., architect, have been approved by the Local Government Board for a fever hospital containing 22 beds.

Colchester.—It has been recommended that the architect to the Infirmary Authorities be instructed to prepare plans of a two-storied building to adjoin the east end of the existing male wards providing for forty additional beds, and that the school-house, containing sixteen beds, be used for consumptive cases.

Gainsborough.—Mr. W. Eyre is the architect for the Coupland Hospital for which some £50,000 was left by Mr. John Coupland, of Hemswell. The foundation stone has just been laid.

Garston.—Towards the cost of the proposed emergency and accident hospital £5,000 is being contributed by the Liverpool Corporation Finance Committee.

Hampstead.—Plans for the erection of a new out-patients' department of the General Hospital in Bayham Street and Greenland Place have been approved by the Borough Council.

Hampstead.—Messrs. Young & Hall, F.R.I.B.A., are architects for the new Out-Patients' Department in connection with the Hampstead General Hospital in Camden Town.

Hinckley.—At the joint hospital meeting Dr. Robinson presented a new plan showing a gain of nine beds by the adoption of the open verandah system The committee decided to recommend the plan for approval.

Hull.—A new sanatorium has been recommended by the hospital sub-committee.

Wigan.—Messrs. W. C. Ralph & Son are designing the proposed new out-patients' department.

### HOSPITAL ARCHITECTURE.

### ANSWERS TO CORRESPONDENTS.

H.J.—Will you please let me know the name of the maker of the Composite Floor as mentioned on page 542 of your issue of January 28?

"Linolite" Flooring Co., Acme Works, Crowes Market, Seven Sisters Road, London, N. The floor analysed is "Doloment." The greatest care should be taken to supervise the laying of this type of flooring, and a guarantee should be insisted on to make good for a number of years; this would tend to ensure careful workmanship.—Ed. The Hospital Architecture Dept.

### THE WEEK'S NOVELTIES.

One of the first meat extracts on the market was that manufactured by the Valentine's Meat-Juice Company, and all that need be said about it is that it has had little difficulty in maintaining its position against all comers. **VALENTINE'S MEAT JUICE** is well-known as a nutriment that usually can be assimilated by the most disordered stomach, and its qualities are invariable.

**FELLOWS' SYRUP OF HYPOPHOSPHITES** has received for a considerable time the attention of expert opinion, and its value in certain cases has often been attested. This syrup contains hypophosphites in a peculiarly useful form; further particulars may be found in our advertisement columns.

The difficult problems of the best way in which invalids can be lifted, without meanwhile disturbing their position, has received several ingenious answers from Mr. Arthur Skeffington. His latest invention, of which illustrated particulars may be found in our advertisement columns, is the **CATHARIOS**, to be run under a hospital bed, for lifting a patient's rectum only. Readers are referred to the illustration for details of its working.

